# Tuberculous Spondylitis: A Report of Different Clinical Scenarios and Literature Update

**DOI:** 10.1155/2017/4165301

**Published:** 2017-12-17

**Authors:** Catarina Lacerda, Rita Linhas, Raquel Duarte

**Affiliations:** ^1^Pulmonology Department, Hospital de Braga, Braga, Portugal; ^2^Pulmonology Department, Centro Hospitalar de Vila Nova de Gaia/Espinho, Vila Nova de Gaia, Portugal; ^3^Chest Disease Centre, Vila Nova de Gaia, Portugal; ^4^Department of Clinical Epidemiology, Predictive Medicine and Public Health, University of Porto Medical School, Porto, Portugal; ^5^EpiUnit, Institute of Public Health, University of Porto, Porto, Portugal

## Abstract

Tuberculosis is still one of the most important health problems in the world. In developed countries, the proportion of extrapulmonary tuberculosis cases is increasing. Nowadays tuberculous spondylitis, also known as Pott disease, is a rare clinical condition but can cause severe vertebral and neurological sequelae that can be prevented with an early correct diagnosis. The aim of this paper is to increase awareness of tuberculous spondylitis in modern times, describing three different cases and discussing its best diagnostic and therapeutic approach based on the current literature.

## 1. Introduction

In 2014, among all 6 million notified cases of tuberculosis (TB), 0.8 million (14%) were new cases of extrapulmonary tuberculosis [1]. Tuberculous spondylitis, also called Pott disease, accounts for 1–5% of TB cases and represents about 50% of all bone and joint TB [2]. Despite the successful achievement in decreasing global pulmonary TB incidence in the last decades, the proportion of extrapulmonary tuberculosis seems to be increasing in developed countries, mainly as a consequence of higher immigration rates and human immunodeficiency virus (HIV) infection [3]. Despite all technological advances, the diagnosis of tuberculous spondylitis remains a clinical challenge since it depends on a high grade of clinical suspicion. Notwithstanding the low reported mortality of tuberculous spondylitis, this condition is still associated with significant clinical morbidity. In particular, significant diagnostic delay may lead to severe skeletal deformities and irreversible neurological complications [4].

The purpose of this paper is to describe three cases of tuberculous spondylitis, focusing the diagnostic and treatment options according to the current literature.

## 2. Case Reports

### 2.1. Case 1

A 37-year-old Portuguese man, heavy smoker, with a history of alcohol and drug abuse, and concomitant HIV infection (CD4^+^T cell count 360 cells/μL) under highly active antiretroviral therapy since 1998, was currently medicated with efavirenz, emtricitabine, and tenofovir. One year before presentation, he had a TB contact and was not screened for active or latent TB infection. He was admitted due to a two-month history of severe back pain, with no constitutional or respiratory symptoms and no neurological signs. Magnetic resonance imaging (MRI) of the spine revealed peripheral lesions located at T9-T10 vertebral bodies, associated with discreet compression of spinal cord, small paravertebral abscess, and empyema. A percutaneous biopsy was performed. The microscopy smear of the acid-fast bacilli (AFB), the nucleic acid amplification test (NAAT), and the culture of *Mycobacterium tuberculosis* (*MT*) were positive, with no drugs resistance. The patient was started on isoniazid (H), rifampin (R), pyrazinamide (Z), and ethambutol (E) (HRZE) for 4 months, due to slow radiological resolution of the vertebral lesions, and remained in therapy with HR for 2 years. He currently has no symptoms or significant vertebral sequelae.

### 2.2. Case 2

**Figure 1:**
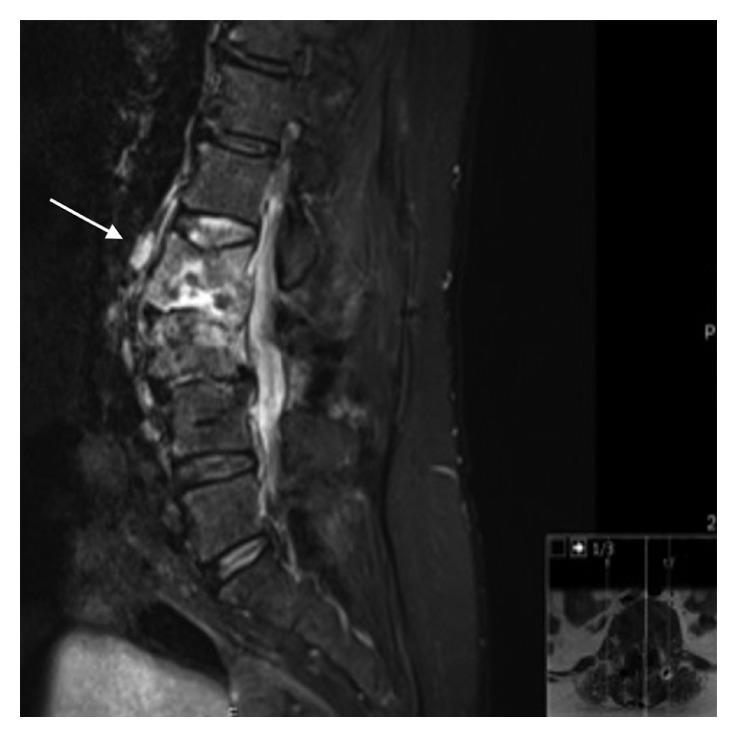
Case 2: sagittal T2-weighted image showing lytic lesions at L2 and L3 vertebral bodies, intervertebral disc destruction, anterior epidural abscess (white arrow), and scoliotic deformity centered in L2-L3.

A 60-year-old Portuguese woman, nonsmoker, HIV negative, with a history of vertebral decompression surgery due to a herniated disc 8 years before, presented with severe back pain lasting 18 months and left lower limb paresthesia, with no fever or respiratory symptoms. MRI of the spine showed central lesions at bodies of L2 and L3, intervertebral foramen reduction, and compression of the L3 spinal root, as well as an epidural abscess (white arrow in Figure 1). The patient had surgery with fixation and arthrodesis of the segments involved. Surgical biopsy revealed *MT* (NAAT and culture positive; no drug resistance). The patient was treated with 2 months of HRZE and then HR until 1 year. She has now chronic back pain controlled with medication, no neurological impairment, or significant kyphotic deformity.

### 2.3. Case 3

A 23-year-old Portuguese man, smoker, HIV negative, with recent multidrug-resistant tuberculosis (MDR-TB) contact, presented with acute lower back pain with radiation to the right flank. There were no respiratory, constitutional, or neurological symptoms. Abdominal computerized tomography (TC) showed a retroperitoneal perirenal abscess. Empiric antibiotic treatment and surgical drainage were performed. After 4 months of persistent symptoms, a MRI of the spine was done, which showed instability of the spine due to total lytic bone destruction of T11 and T12 vertebral bodies, focal lytic lesions at bodies T5-T10 bodies, perivertebral abscesses, and also a psoas abscess. A percutaneous biopsy revealed *MT* (AFB and culture positive). Molecular testing for drug resistance was positive to isoniazid and rifampicin. No other resistance was observed in culture with drug sensibility tests. Other organ involvement was excluded. Surgical stabilization with an autograph reconstruction of T11 and T12 was performed. The patient started an anti-MDR TB therapy with pyrazinamide, ethambutol, amikacin, levofloxacin, ethionamide, and cycloserine. He finished treatment after two years. He has now a moderate chronic back pain and vertebral body loss although with no kyphotic deformity.

## 3. Discussion

Three distinct scenarios of tuberculous spondylitis were described, enhancing different clinical characteristics, treatment approaches, and outcomes.

Commonly, in tuberculous spondylitis, the symptoms develop insidiously due to the slow progression of the disease, contributing to a significant delay between symptoms onset and diagnosis. Even in developed countries, as we observed in the cases described, time to diagnosis can take more than six months, and it represents one of the worst prognostic factors [5]. Back pain is the most common symptom (83–100% of the patients), and constitutional symptoms, including fever, are relatively rare (33%) [3]. Spine deformities and neurological deficits are the worst complications of tuberculous spondylitis. Despite all the advances in diagnostic techniques, in developed countries, neurological deficits are still present at the time of diagnosis in 45% of the cases [6].

The mean age of patients with tuberculous spondylitis is 45–60 years, although two peaks are reported concerning risk factors: one between 20 and 30 years, related to immigration and HIV, and one between 60 and 70 years, related to immunosuppression and comorbidities [5]. In case 2, the older age and the inexistence of constitutional symptoms or major risk factors may have contributed to the longer delay in the diagnosis. In these situations, other more frequent diagnoses of chronic back pain are usually considered, such as degenerative joint disease. In the other hand, cases with more acute onset of the symptoms may raise the hypothesis of pyogenic infection [7] In cases 1 and 3, patients were young and had associated major risk factors (HIV and previous TB/MDR-TB contact), which made the hypothesis of TB infection more likely.

Commonly, tuberculous spondylitis affects the thoracic and thoracolumbar segments with initial destruction of the vertebral bodies. Other segments and multifocal involvement are uncommon [5]. Concomitant paraspinal abscesses and epidural involvement can be seen, respectively, in around 70% and 65% of the cases. As in the cases described, a significant percentage of patients do not show evidence of the primary infection focus. Concurrent pulmonary involvement can range from 2 to 65% [3].

Diagnostic suspicion of tuberculous spondylitis is based on clinical and radiological features. Spinal radiography may show a destructive process of vertebras and adjacent discs if osteomyelitis is present. These findings only appear in a late course of the disease and are less pronounced compared to pyogenic infection. Nowadays, highly sensitive and specific techniques are available that can overcome these limitations. Spinal CT and MRI can achieve the same diagnostic yield [5]. The CT helps to define the extent of the disease and is the best method to detect calcified foci, characteristic in tuberculous infections and rare in pyogenic infections. Spinal MRI is more sensitive in early stages of the disease since it provides better tissue contrast than CT and permits a better visualization of the epidural space and spinal cord. The overall sensitivity and specificity of MRI for tuberculous spondylitis is 100% and 80%, respectively, which renders the MRI the best radiological method for the diagnosis of tuberculous spondylitis [8]. MRI is also helpful to clarify the need for surgical intervention, since it is the most precise radiological method to assess nervous system involvement and spine instability.

Although clinical and radiological findings can be suggestive of tuberculous spondylitis, biopsy—either open biopsy or CT-guided percutaneous aspiration biopsy (PAB)—should be performed in order to obtain a definitive diagnosis. PAB has a low morbidity associated and a diagnostic yield of 68%, which makes it the method of choice in patients with no indication to surgery [9]. The tuberculin skin test (TST) and interferon-gamma release assays (IGRAs) are not routinely used in the diagnosis of extrapulmonary TB since they cannot differentiate latent from active TB infection. However, in difficult cases, their high negative predictive value may be helpful in rule out tuberculous spondylitis in the presence of spinal osteomyelitis. When tuberculous spondylitis coexists with other forms of TB, if MT is already documented in other specimen and typical radiological features of tuberculous spondylitis are present, we can assume the same etiology; otherwise, MT should be isolated to confirm the diagnosis. Microbiological analyses can include acid-fast bacilli (AFB) microscopy smear, the nucleic acid amplification test (NAAT), culture identification of MT, and the drug susceptibility test (DST). Histological analysis should also be performed since the presence of caseating granulomas can support the diagnosis. The extrapulmonary samples (EPS) are characteristically paucibacillary which difficult the diagnosis. Diagnostic sensitivity of AFB smear microscopy (Ziehl–Neelsen and fluorescent staining) is low, although in some studies it can be seen in up to 58% of the cases [9, 10]. Sensitivity of MT culture can be as high as 83% in solid media (Lowenstein-Jensen), but the slow growing time of up to 3–8 weeks is a limitation [11]. Liquid media, such as Middlebrook media, have been developed in order to reduce the mean time of culture (2-3 weeks) and also show higher sensitivity [9]. MT culture is necessary for an accurate DST in order to obtain the best therapeutic regimen. Despite the fact that paucibacillar forms and NAAT inhibitors contribute to false negative results, several studies show that NAAT can be successfully tested in EPS, achieving a sensitivity of 72% [12]. This method can reduce the diagnosis time to hours, and therefore, it is also helpful to readily differentiate between MT and no MT infection. However, international guidelines still only recommend this test in respiratory specimens. In case 1, positive AFB microscopy smear and NAAT allowed to make an early definitive diagnosis of tuberculous spondylodiscitis. In case 2, a negative AFB and positive NAAT made the diagnosis of MT infection more likely, avoiding a delay in the initiation of anti-TB therapy. More recently, new molecular methods are available for MT identification and drug susceptibility testing. The Xpert MTB/RIF assay has a sensitivity and specificity of 81% and 99%, respectively, in extrapulmonary TB diagnosis and can simultaneously access the resistance to rifampicin within two hours [13]. Specifically in bone and joint TB, the Xpert MTB/RIF assay demonstrated a sensibility of 82% and specificity of 100% in the diagnosis and a 100% concordance with culture-DST for the detection of RIF resistance [14]. This assay was endorsed for extrapulmonary TB diagnosis by the WHO in 2013 [15]. Other tests such as the GenoType MTBDRplus (MTBDR) assay, which detects both rifampicin and isoniazid resistances, have been also studied, showing sensitivity of 72% in bone and joint TB, although more data are needed [14]. These molecular tests provide a rapid access to results and consequently an early correct first-line treatment. In cases of suspected MDR, as case 3, Xpert should be performed.

The goal of tuberculous spondylitis treatment is not only to eradicate infection but also to treat and prevent neurological complications and spinal deformities. Pharmacological treatment should be initiated as soon as the diagnosis is confirmed, with 2 months of HRZE (intensive phase) followed by 4 to 7 months of HR (continuation phase). The duration of treatment remains controversial. Due to difficulties in assessing response and risk of relapse, most experts recommend 9 to 12 months of treatment, and in situations of slow radiological resolution as case 2, 12 to 24 months of treatment should be considered [16–18]. The hypothesis of MDR must always be taken into account, and a definitive anti-TB regimen should be based on susceptibility tests. MDR is usually a result of inappropriate drug therapy and is rarely innate [19]. In case 3, his previous contact raised the possibility of a MDR-TB, and thus the anti-TB regimen was based on susceptibility test of the contact patient. Directly observed treatment (DOT) is recommended in order to ensure treatment adherence. Corticosteroid therapy is not recommended in cases of tuberculous spondylitis unless there is meningeal involvement. Surgery is confined to patients that present with neurological deficits caused by spinal cord compression, spinal deformity with instability, severe or progressive kyphosis, large paraspinal abscesses, and no response or failure of anti-TB therapy [6]. As described, both cases 2 and 3 had clear indication for surgery. In contrast, the clinical and radiological features of case 1 were indicative for conservative treatment. Conservative treatment is considered in cases with mild to moderate neurologic deficits, mild vertebral body involvement (one central lesion or various peripheral lesions in up to 3 vertebral bodies) and canal involvement, mild paravertebral abscess, no retropharyngeal or psoas abscess, and no significant kyphosis (less than 30°) or intervertebral instability [18]. The need for surgery is directly related to the diagnostic delay, and it is seen in more than 50% of the cases [20]. Indication for surgery also depends on available local resources and surgical expertise. The pharmacological and surgical treatments, when necessary, are effective and have a low relapse rate (0–5%). Studies in the last decade showed a mortality rate of 4% [3].
